# Association between person-centered maternity care and newborn complications in Kenya

**DOI:** 10.1002/ijgo.12978

**Published:** 2019-09-23

**Authors:** May Sudhinaraset, Amanda Landrian, Patience A. Afulani, Nadia Diamond-Smith, Ginger Golub

**Affiliations:** 1 Jonathan and Karin Fielding School of Public Health, University of California, Los Angeles (UCLA), Los Angeles, CA, USA; 2 School of Medicine, Institute for Global Health Sciences, University of California, San Francisco (UCSF), San Francisco, CA, USA; 3 Innovations for Poverty Action, Nairobi, Kenya

**Keywords:** Health-seeking behaviors, Kenya, Neonatal health, Person-centered maternity care, Quality of care

## Abstract

**Objective:**

Despite the recognized importance of person-centered care, very little information exists on how person-centered maternity care (PCMC) impacts newborn health.

**Methods:**

Baseline and follow-up data were collected from women who delivered in government health facilities in Nairobi and Kiambu counties in Kenya between August 2016 and February 2017. The final analytic sample included 413 respondents who completed the baseline survey and at least one follow-up survey at 2, 6, 8, and/or 10 weeks. Data were analyzed using descriptive, bivariate, and multivariate statistics. Logistic regression was used to assess the relationship between PCMC scores and outcomes of interest.

**Results:**

In multivariate analyses, women with high PCMC scores were significantly less likely to report newborn complications than women with low PCMC scores (adjusted odds ratio [aOR] 0.39, 95% confidence interval [CI] 0.16–0.98). Women reporting high PCMC scores also had significantly higher odds of reporting a willingness to return to the facility for their next delivery than women with low PCMC score (aOR 12.72, 95% CI 2.26–71.63). The domains of Respect/Dignity and Supportive Care were associated with fewer newborn complications and willingness to return to a facility.

**Conclusion:**

PCMC could improve not just the experience of the mother during childbirth, but also the health of her newborn and future health-seeking behavior.

## 1 | INTRODUCTION

Despite major gains in the past decade, maternal and newborn deaths remain unacceptably high. Every year, 303 000 women die of pregnancy and childbirth-related complications, while 2.6 million newborns die in the first month of life worldwide.^1^ Poor quality of care is a major factor in maternal and newborn health, with both short-and long-term effects on women and families.^2,3^ Recent evidence of poor treatment of women during childbirth has increased attention to aspects of quality beyond clinical or essential services. This has led to calls for greater focus on person-centered reproductive health care: care that is respectful of and responsive to women’s and families’ preferences, needs, and values.^4^ Despite the recognized importance of person-centered care, little research exists on how person-centered maternity care (PCMC) impacts newborn health, such as neonatal complications and immunizations.

A recent systematic review on interventions to improve PCMC, such as continuity of midwifery care, decision-making tools, and information provision on various outcomes (including maternity care, perinatal health, and mental health outcomes) found mixed results regarding the impact of PCMC interventions on labor and delivery outcomes.^5^ There was also poor evidence for the impact of these interventions on perinatal-related outcomes.^5^ Outside of maternity care, however, person-centered care approaches in primary care settings are associated with reduced specialty care clinic visits, hospitalizations, and healthcare costs.^6^ More evidence is therefore needed on how PCMC impacts health outcomes, including newborn outcomes.

Quality of care is particularly relevant in Kenya, where 90% of maternal deaths at major referral hospitals in 2017 resulted from substandard care.^6^ Studies have also documented poor PCMC in Kenya. One study found that on a scale of 0–100, PCMC was less than 70% in Kenya.^7^ In addition, one in five women reported feeling humiliated during labor and delivery.^8^ Despite the documented evidence of poor PCMC, there is little evidence on its consequences. A major reason for this gap in the literature is the lack of validated measures for PCMC and clinical follow-up data linking health outcomes to women’s experiences of care. Past literature is expanded upon by using data on a validated measure of PCMC^4^ and follow-up data on health outcomes at 2–10 weeks postpartum among women in Kenya.

The primary aim of the present study was to explore the association between PCMC and newborn-related outcomes in Kenya, including newborn complications and rates of immunization. A secondary aim was to examine the association between PCMC and a woman’s intention to deliver in the same facility in the future. It was hypothesized that women who experience higher levels of PCMC will report lower levels of newborn complications, higher newborn immunizations, and greater willingness to deliver in the same facility for her next delivery.

## 2 | MATERIALS AND METHODS

### 2.1 | Study participants and recruitment

Respondents were recruited from seven government health facilities in Nairobi and Kiambu counties in Kenya. Baseline and follow-up surveys were completed between August 2016 and February 2017. Participants were recruited during their recovery in the post-partum ward and were eligible to participate if they were aged 15–49 years, had a normal delivery (i.e. not scheduled cesarean delivery) within the last 7 days, possessed a text-equipped phone, and reported feeling well enough to participate. In total, 531 respondents completed the baseline survey, while 62 refused participation and 124 were ineligible due to not having their own text-equipped phone or not delivering in the past 2 weeks. Written informed consent was obtained before conducting study procedures.

Baseline respondents received airtime credit worth approximately US$1.00 for their participation. Follow-up surveys took approximately 10 minutes and were conducted using mSurvey, in which questions were sent via phone and respondents answered through free text messages. Respondents received follow-up surveys at 2, 6, 8, and 10 weeks after baseline. Participants received $0.20 in airtime credit for completion of each follow-up survey.

### 2.2 | Survey measures

Dependent variables: The primary outcomes of interest were newborn complications, visiting a health facility for newborn immunizations, and women’s willingness to deliver in the same facility for her next delivery. Newborn complications were assessed at a 2-week follow-up by asking women to report whether their newborn had experienced any health complications, including jaundice, fever, difficulty breathing, or other problems. Having visited a health facility for the newborn’s immunizations was assessed at the 6-week and 10-week follow-ups; indicating “Yes” at either follow-up was defined as a newborn having received immunizations in the newborn period. The mother’s willingness to return to the same facility for her next delivery was assessed at the 8-week follow-up.

Independent variables: The key independent variable was a validated 30-item PCMC scale (Cronbach’s α=0.84) comprising three sub-scales: Dignity and Respect (six items); Communication and Autonomy (nine items); and Supportive Care (15 items).^4^ This was measured at baseline directly after women’s childbirth experience. For example, respondents were asked: “How did you feel about the amount of time you waited?” “Did the doctors, nurses, or other staff at the facility treat you with respect?” “Do you think there was enough health staff in the facility to care for you?” Response options for each item range from 0 (“No, never”) to 3 (“Yes, all the time”). Responses were totaled across all 30 items to obtain a total PCMC score in the range of 0–90, where higher scores indicate better PCMC. Total scores were also obtained for each sub-scale with scores in the range of 0–18 for Dignity and Respect, 0–27 for Communication and Autonomy, and 0–45 for Supportive Care. Four variables were then created categorizing total PCMC scores, as well as each sub-scale score, as “low,” “medium,” or “high,” with scores in the approximate lower 25th percentile defined as “low” and scores in the top 75th percentile defined as “high.”

A summary measure of postpartum care included 12 items across three sub-indices: facility-level characteristics of the postnatal ward (e.g. availability of a separate bed, food, water supply), receipt of postpartum health checks (e.g. providers checking on the woman’s general health), and postpartum counseling (e.g. advising the woman about exclusive breastfeeding, how to properly bathe the baby). Response options for each item were 0 (“No”) and 1 (“Yes”) and were totaled to obtain a postpartum quality of care score in the range of 0–12, where higher scores indicate better quality of care. A variable was created which categorized total scores as either “low,” “medium,” or “high” postpartum quality of care received using the same percentile cutoffs as PCMC above.

In addition, we included information on sociodemographic characteristics, including current age, number of births, marital status, educational attainment, employment status, whether they were born in Nairobi or Kiambu County (as an indicator of internal migration), and whether they were covered under a health scheme or health insurance, as well as self-rated health status and experience of maternal complications during labor or delivery.

### 2.3 | Analyses

**FIGURE 1:**
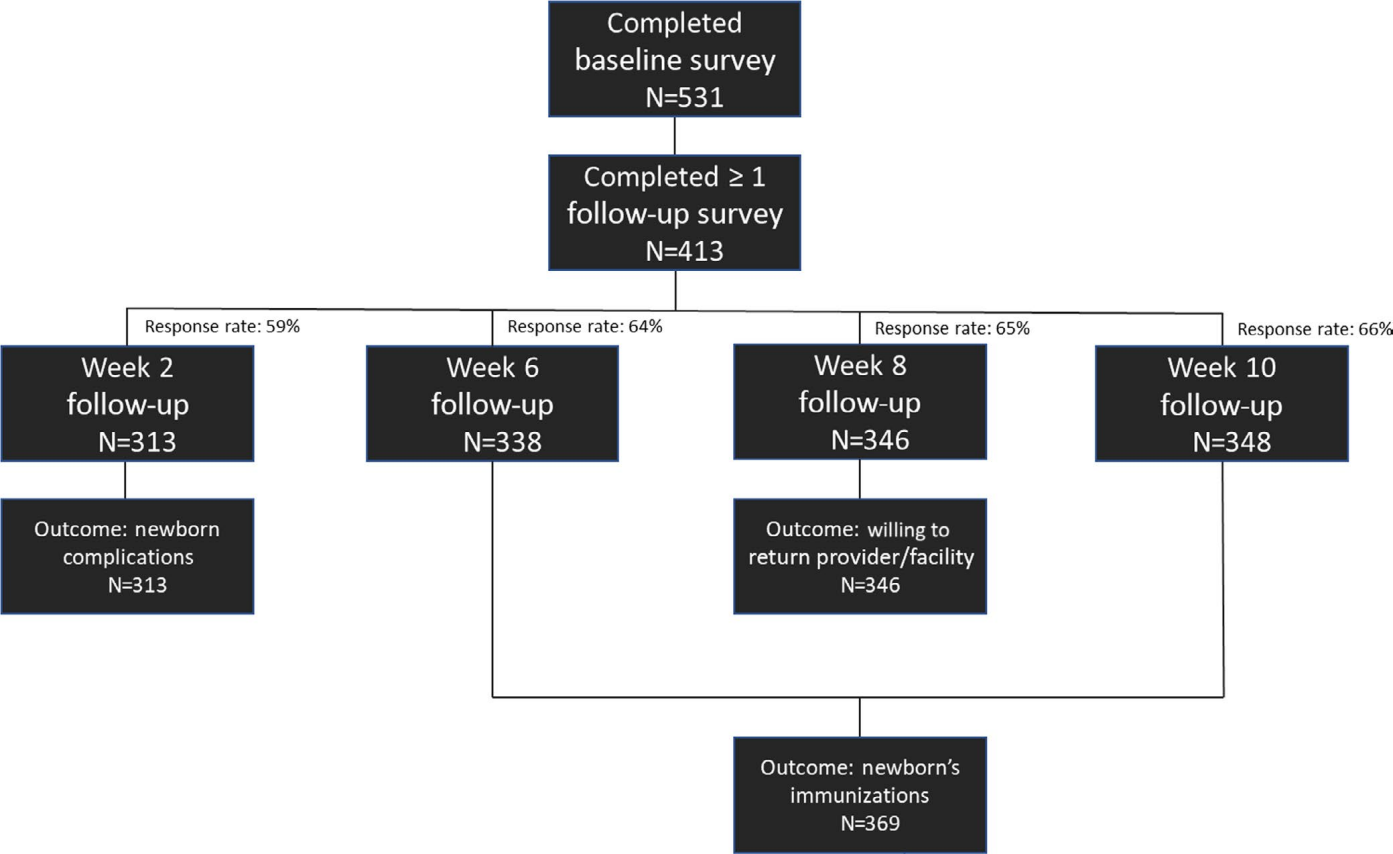
Flow chart of baseline and follow-up sample sizes and response rates.

Data were analyzed with descriptive, bivariate, and multivariate statistics using StataSE version 15.^9^ Bivariate and multivariate analyses were completed using women who had data at baseline and completed at least one follow-up survey (i.e. had valid data for at least one outcome of interest). A total of 413 women had complete data at baseline and at least one follow-up survey. Figure 1 illustrates baseline and follow-up rates and the number of respondents with complete data on newborn complications, newborn’s immunizations, and willingness to return to the facility. Nearly 60% (n=244, 59%) of respondents had complete information on all outcomes of interest (i.e. completed all follow-up surveys).

Logistic regression was used to assess the relationship between PCMC score (measured at baseline) and each of the dependent variables of interest (measured at follow-up). Logistic regression analyses were also used to assess the association between each PCMC sub-scale and each of the dependent variables of interest, respectively. All multivariate models controlled for sociodemographic characteristics, postpartum quality of care, and experience of maternal complications during labor or delivery.

The Institutional Review Boards at the University of California, San Francisco and Kenya Medical Research Institute approved all study procedures.

## 3 | RESULTS

Sociodemographic characteristics of the study sample (n=413) are presented in Table 1. Over one-third (31.1%) of women reported their newborn experienced complications within 2 weeks of delivery (Table 2). Most women returned to the facility for their newborn’s immunizations (89.7%) and were willing to return to the same facility for their next delivery (85.0%) (Table 2).

**TABLE 1:** Sociodemographic characteristics and postpartum quality of care received during delivery (n=413).

Characteristic	N (%) or mean (SD)^a^
Age (years)	25.5 (4.8)
Number of births	2.2 (1.1)
Multiparous	
No	131 (31.7)
Yes	282 (68.3)
Currently married or partnered	
No	64 (15.5)
Yes	349 (84.5)
Reading ability	
Able, without difficulty	392 (94.9)
Able, with some difficulty	20 (4.8)
Unable	1 (0.2)
Writing ability	
Able, without difficulty	396 (95.9)
Able, with some difficulty	15 (3.6)
Unable	2 (0.5)
Highest level of education completed	
Primary or less	150 (36.3)
Vocational/secondary	191 (46.3)
College/University	72 (17.4)
Employed	
No	202 (48.9)
Yes	211 (51.1)
Religion	
Protestant	248 (60.1)
Catholic	101 (24.5)
Other Christian	60 (14.5)
Muslim	3 (0.7)
None	1 (0.2)
Born in Kiambu or Nairobi County	
No	298 (72.2)
Yes	115 (27.9)
Covered under health scheme or health insurance	
No	267 (64.7)
Yes	146 (35.4)
Current self-rated health	
Excellent or very good	45 (10.9)
Good	247 (59.8)
Fair	101 (24.5)
Poor or very poor	20 (4.8)
Postpartum quality of care received	
Low	102 (24.7)
Medium	189 (45.8)
High	122 (29.5)
Maternal complications during labor or delivery	
No	323 (78.2)
Yes	90 (21.8)

a Values are given as number (percentage) or mean (standard deviation). Percentages may not add up to 100 due to rounding.

**TABLE 2:** Total PCMC and sub-scale scores at delivery and newborn and health-seeking behavior-related outcomes measured at follow-up.

Outcome	n (%)^a^
Total PCMC Score	n=413
Low	96 (23.2)
Medium	213 (51.6)
High	104 (25.2)
Dignity and Respect sub-scale score	n=413
Low	100 (24.2)
Medium	159 (38.5)
High	154 (37.3)
Communication and Autonomy sub-scale score	n=413
Low	95 (23.0)
Medium	174 (42.1)
High	144 (34.9)
Supportive Care sub-scale score	n=413
Low	97 (23.5)
Medium	212 (51.3)
High	104 (25.2)
Newborn experienced complications within 2 wk of delivery^b^	n=313
No	215 (68.7)
Yes	98 (31.3)
Returned to the facility for newborn's immunizations	n=369
No	38 (10.3)
Yes	331 (89.7)
Would return to the same provider/facility next time delivering a baby	n=346
No	52 (15.0)
Yes	294 (85.0)

Abbreviation: PCMC, person-centered maternity care.

a Percentages may not add up to 100 due to rounding.

b Newborn complications included: jaundice (n=30); fever (n=14); difficulty breathing (n=14); difficulty breastfeeding (n=10); rash (n=9); seizure (n=1); and other (n=20).

In bivariate analysis, women with high PCMC scores had significantly lower odds of reporting newborn complications within 2 weeks than women with low PCMC scores (odds ratio [OR] 0.39, 95% confidence interval [CI] 0.20–0.78) (Table 3). Women with high PCMC scores had significantly higher odds of reporting a willingness to return to the same facility for their next delivery than women with low PCMC scores (OR 12.29, 95% CI 3.53–42.73). PCMC scores were not significantly associated with visiting a facility for the newborn’s immunizations (Table 3).

**TABLE 3:** Logistic regressions examining the bivariate relationship between PCMC and newborn and health-seeking behavior-related outcomes.^a^

Characteristic	Newborn experienced complications within 2 wk of delivery (n=313)	Visited facility for newborn’s immunizations (n=369)	Willing to return provider/facility for next delivery (n=346)
PCMC score			
Low	Ref	Ref	Ref
Medium	0.55 (0.31–0.96)^a^	1.55 (0.69–3.48)	2.61 (1.38–4.93)^b^
High	0.39 (0.20–0.78)^b^	1.31 (0.53–3.27)	12.29 (3.53–42.73)^c^

Abbreviation: PCMC, person-centered maternity care.

a Values are given as adjusted odds ratios (95% confidence interval). Model sample sizes vary based on women who had complete data at baseline and who reported the respective outcome.

b *P*<0.05.

c *P*<0.01.

d *P*<0.001.

In multivariate logistic regressions, higher PCMC scores were negatively associated with newborn complications within 2 weeks after delivery (Table 4). Controlling for other covariates, women with medium and high PCMC scores had significantly lower odds of reporting newborn complications than women with low PCMC scores (adjusted OR [aOR] 0.50, 95% CI 0.28–0.92 and aOR 0.38, 95% CI 0.18–0.81, respectively). Women with medium and high PCMC scores also had significantly higher odds of reporting a willingness to return to the same facility for their next delivery than women with low PCMC scores (aOR 2.88, 95% CI 1.46–5.69 and aOR 14.11, 95% CI 3.81– 52.28, respectively). PCMC scores were not significantly associated with visiting a facility for the newborn’s immunizations. Postpartum quality of care was found to be marginally predictive of visiting a facility for the newborn’s immunizations. None of the other covariates included in the models were found to be significantly associated with the outcomes of interest.

**TABLE 4:** Multivariate logistic regressions examining the relationship between PCMC and newborn and health-seeking behavior-related outcomes.

Characteristic	Newborn experienced complications within 2 wk of delivery (n=313)	Visited facility for newborn’s immunizations (n=369)	Willing to return provider/facility for next delivery (n=346)
PCMC score			
Low	Ref	Ref	Ref
Medium	0.50 (0.28–0.92)^b^	1.51 (0.65–3.52)	2.88 (1.46–5.69)^c^
High	0.38 (0.18–0.81)^b^	1.15 (0.42–3.19)	14.11 (3.81–52.28)^d^
Age (years)	0.94 (0.87–1.01)	0.92 (0.84–1.00)	0.97 (0.88–1.05)
Married			
No	Ref	Ref	Ref
Yes	1.18 (0.58–2.41)	1.53 (0.59–3.96)	1.08 (0.45–2.64)
Educational attainment			
Primary or less	Ref	Ref	Ref
Vocational/secondary	0.85 (0.47–1.56)	1.20 (0.54–2.68)	0.62 (0.30–1.31)
College/University	1.16 (0.54–2.51)	0.84 (0.30–2.38)	0.52 (0.19–1.45)
Employed			
No	Ref	Ref	Ref
Yes	1.12 (0.65–1.93)	0.67 (0.31–1.44)	0.78 (0.39–1.56)
Born in Kiambu or Nairobi County			
No	Ref	Ref	Ref
Yes	0.93 (0.53–1.64)	1.52 (0.65–2.54)	1.51 (0.71–3.21)
Covered under health scheme or health insurance			
No	Ref	Ref	Ref
Yes	1.43 (0.82–2.51)	1.51 (0.68–3.38)	1.74 (0.82–3.70)
Multiparous			
No	Ref	Ref	Ref
Yes	0.69 (0.34–1.40)	1.26 (0.45–3.56)	0.63 (0.25–1.60)
Self-rated health status			
Fair or poor	Ref	Ref	Ref
Excellent, very good, or good	0.77 (0.44–1.36)	0.59 (0.24–1.45)	1.34 (0.63–2.86)
Postpartum quality of care received			
Low	Ref	Ref	Ref
Medium	0.97 (0.51–1.85)	1.77 (0.79–4.00)	1.93 (0.91–4.09)
High	0.80 (0.39–1.63)	2.64 (1.01–6.88)^b^	1.53 (0.67–3.49)
Maternal complications during labor or delivery			
No	Ref	Ref	Ref
Yes	1.68 (0.93–3.03)	1.35 (0.55–3.33)	1.45 (0.65–3.27)

Abbreviation: PCMC, person-centered maternity care.

a Values are given as adjusted odds ratios (95% confidence interval). Model sample sizes vary based on women who had complete data at baseline and who reported the respective outcome.

b *P*<0.05.

c *P*<0.01.

d *P*<0.001.

In multivariate logistic regressions, with the PCMC sub-scale scores as the key predictors and controlling for other variables, the PCMC sub-scale scores were associated with the various outcomes. Compared to women with low scores on the Dignity and Respect sub-scale, high scores on the Dignity and Respect sub-scale were associated with significantly lower odds of newborn complications at 2 weeks after delivery (aOR 0.49, 95% CI 0.25–0.94) and significantly higher odds of women reporting a willingness to return to the facility for their next delivery (aOR 5.58, 95% CI 2.39–13.01) (Table 5). Additionally, compared to low scores on the Supportive Care sub-scale, high scores on the Supportive Care sub-scale were associated with significantly lower odds of newborn complications at 2 weeks after delivery (aOR 0.38, 95% CI 0.18–0.81). Medium or high scores in the Supportive Care sub-scale were also associated with significantly higher odds of reporting a willingness to return to the facility for their next delivery (aOR 2.63, 95% CI 1.35–5.14 and aOR 43.75, 95% CI 5.44–351.59, respectively). Scores on the Communication and Autonomy sub-scales were only associated with women’s reported willingness to return to the same facility for their next delivery (aOR 2.16, 95% CI 1.05–4.45 and aOR 3.71, 95% CI 1.59–8.66, respectively for medium and high scores on the Communication and Autonomy sub-scales). None of the PCMC sub-scales were found to be associated with visiting a facility for the newborn’s immunizations.

**TABLE 5:** Multivariate logistic regressions examining the relationship between PCMC sub-scales and newborn and health-seeking behavior- related outcomes.^a^

PCMC sub-scale score^b^	Newborn experienced complications within 2 wk of delivery (n=313)	Visited facility for newborn’s immunizations (n=369)	Willing to return provider/facility for next delivery (n=346)
Dignity and Respect			
Low	Ref	Ref	Ref
Medium	0.69 (0.37–1.29)	0.99 (0.39–2.50)	3.68 (1.74–7.77)^d^
High	0.49 (0.25–0.94)^c^	1.00 (0.39–2.60)	5.58 (2.39–13.01)^e^
Communication and Autonomy			
Low	Ref	Ref	Ref
Medium	0.83 (0.44–1.58)	1.30 (0.53–3.19)	2.16 (1.05–4.45)^c^
High	0.72 (0.37–1.43)	1.04 (0.41–2.62)	3.71 (1.59–8.66)^d^
Supportive Care			
Low	Ref	Ref	Ref
Medium	0.61 (0.33–1.12)	1.72 (0.78–3.80)	2.63 (1.35–5.14)^e^
High	0.38 (0.18–0.81)^c^	1.94 (0.99–10.17)	43.75 (5.45–351.59)^e^

Abbreviation: PCMC, person-centered maternity care.

a Values are given as adjusted odds ratios (95% confidence interval).

b Each model controls for: age, marital status, educational attainment, employment status, whether they were born in Kiambu or Nairobi County, health insurance coverage, parity, self-rated health status, postpartum quality of care received, and experience of maternal complications. Sample sizes for each model varies based on women who had complete data at baseline and who reported that respective outcome at follow-up.

c *P*<0.05.

d *P*<0.01.

e *P*<0.001.

## 4 | DISCUSSION

To our knowledge, this is the first study to assess the impact of PCMC on newborn outcomes using a validated scale for person-centered care. Although PCMC should be considered a right, and therefore provided whether or not it improves outcomes, the finding that receipt of high PCMC is associated with neonatal health will advance advocacy efforts to improve PCMC.

Perhaps the most intriguing finding in this study was that PCMC was associated with a decrease in reported complications in newborns, particularly for the domains of Respect and Dignity and Supportive Care. It is plausible that PCMC improves neonatal health through increased safety and adherence.^10^ In addition, women who experience better PCMC may have greater trust in their providers and be more likely to follow their advice. PCMC may also be indicative of broader health facility environment factors that improve clinical care. These same mechanisms could be at play for women’s intentions around future care-seeking—women who receive counseling and information and are involved in decision-making regarding care may be more satisfied with their provider and the health facility in general. All three sub-domains of PCMC, including Communication and Autonomy, were associated with women’s intentions around future care-seeking. Future studies should attempt to disentangle mechanisms for PCMC and health outcomes.

While PCMC is associated with improvements in newborn health and women’s intentions for future delivery, there is no significant association with newborn immunizations. Other factors beyond women’s experiences, such as access to care or geographical proximity to the facility, may be more important for returning to a health facility for immunizations.^11^ Additionally, immunizations are likely to take place in a different location from maternal care, hence they may not be influenced by the experience in the birthing facilities.

There are several limitations to this study. First, newborn complications are self-reported by women and it is unclear whether women can accurately recognize newborn complications which might result in misreporting. Second, as women were asked to report PCMC while in the facility, social desirability bias may be present. One study found that women underreported mistreatment in a facility compared to direct observation.^12^ Third, because only women with complete cases at baseline and follow-up were included, the sample in the present study was limited in terms of the capability of the statistical power to detect differences. Loss to follow-up is another limitation: only 46% of women from baseline responded to all follow-up surveys. In assessing differences between those with and without valid data at each follow-up using Pearson *χ*^2^ tests, significant differences were detected in marital status and education at week 2; however, no significant differences in sociodemographic characteristics or PCMC score were detected at weeks 6, 8, and 10. This suggests that estimates are not highly biased by missing data. Lastly, the eligibility criteria of needing a text-equipped phone may have slightly biased the sample in the present study towards wealthier, literate women.

These results have a number of programmatic and policy implications. First, Kenya has recently made great strides in improving newborn care, including making explicit standards for nursing care in newborn units,^13^ as well as passing a unified policy to guide efforts for improving newborn health. A comprehensive strategy that promotes universal access to antenatal care and early postnatal care and a person-centered care approach in maternity settings should be at the forefront of standards to improve newborn and child health.^14^ Second, providers and facility staff should be trained on PCMC. This study may be used to gain buy-in from staff to adopt person-centered care strategies in their facilities. Future research should assess the impact of PCMC on other health outcomes, including maternal complications, breastfeeding practices, and follow-up visits. Every woman should be treated with dignity and respect from a rights-based perspective. These findings will, however, facilitate PCMC advocacy from a health benefits perspective: that PCMC could improve not just the experience of women but also the health of her newborn.
